# Two-Years Prospective Follow-Up Study of Subacute Thyroiditis

**DOI:** 10.3389/fendo.2020.00047

**Published:** 2020-02-28

**Authors:** Na Zhao, Shuo Wang, Xue-Jiao Cui, Ming-Shi Huang, Shi-Wei Wang, Yu-Ge Li, Lei Zhao, Wei-Na Wan, Yu-Shu Li, Zhong-Yan Shan, Wei-Ping Teng

**Affiliations:** ^1^Liaoning Provincial Key Laboratory of Endocrine Diseases, Department of Endocrinology and Metabolism, Institute of Endocrinology, The First Affiliated Hospital of China Medical University, Shenyang, China; ^2^Department of Ultrasonography, The First Affiliated Hospital of China Medical University, Shenyang, China

**Keywords:** subacute thyroiditis, thyroid volume, hypothyroidism, TSH, ultrasonography

## Abstract

**Purpose:** The aim of the present prospective follow-up study was to explore the early indicators of hypothyroidism and the final changes in thyroid volume in subacute thyroiditis (SAT) patients.

**Methods:** We enrolled 61 SAT patients and followed them up for 2 years to assess the incidence of hypothyroidism and changes in thyroid volume. Binary logistic regression and receiver operating characteristic (ROC) curves were used for data analysis.

**Results:** During the 2 years follow-up period, we found that the volumes of the thyroid gland in SAT patients at 1 and 2 years were significantly smaller than those in the healthy control group, which were significantly smaller compared to the initial thyroid volumes after SAT onset (*p* < 0.001). Also, the thyroid volumes of SAT patients with hypothyroidism were significantly smaller than those of SAT patients without hypothyroidism. The early maximum thyroid-stimulating hormone (TSH) values (within 3 months after SAT onset) were closely related to the incidence of hypothyroidism at 2 years. The OR value was 1.18 (95% CI = 1.01–1.38, *p* = 0.032). The early maximum TSH value had a maximum area under the ROC curve (AUC) of 0.866 for the development of hypothyroidism 2 years after SAT onset *vs*. euthyroidism (*p* < 0.001).

**Conclusions:** The thyroid volumes of patients increased significantly after the onset of SAT, while during the follow-up these volumes decreased; the thyroid volumes at 1 and 2 years were significantly smaller than those of normal healthy subjects, especially in SAT patients with hypothyroidism. Furthermore, the early maximum TSH value could be used as an effective indicator of the development of hypothyroidism 2 years after the onset of SAT.

## Introduction

Subacute thyroiditis (SAT), also called subacute granulomatous thyroiditis, de Quervain's thyroiditis, or giant-cell thyroiditis, is the most common cause of anterior cervical pain. Other symptoms, such as fever and radiating pain to the pharynx and jaw, are also observed (1). SAT is a self-limited inflammatory thyroid disease possibly caused by viral infection. The typical indicators or features of SAT include disordered thyroid function, elevated erythrocyte sedimentation rate (ESR) and C-reactive protein (CRP), low thyroid uptake of radioactive iodine or technetium-99m because of the destructive etiology of the hyperthyroidism, and a hypoechoic, irregular lesion in the thyroid lobe on thyroid ultrasonography (US) (2–5). The initial treatment is intended to reduce the inflammation and relieve the symptoms and signs. Unfortunately, some patients develop permanent hypothyroidism (6). Although the clinical features and incidence of SAT have been researched and discussed in many studies (2, 6, 7), prospective follow-up studies providing evidence regarding the prediction of progression to hypothyroidism and final changes in the thyroid volumes of SAT patients have been scarce.

Sixty-one outpatients with SAT in our hospital were followed up for 2 years. The course and outcome of SAT were evaluated. Factors such as the early maximum TSH value, maximum free T4 (FT4), maximum free T3 (FT3), erythrocyte sedimentation rate, and treatment plan were observed to explore the risk factors that may affect thyroid function and imaging changes.

## Materials and Methods

### Participants

From 2015 to 2017, 73 SAT patients without a past medical history of autoimmune thyroid diseases [including Graves disease and autoimmune thyroiditis (AIT)] who consecutively visited the Department of Endocrinology and Metabolism of The First Affiliated Hospital of China Medical University were enrolled. The thyroid volume within 3 months after SAT onset was regarded as the initial thyroid volume. Since we could not obtain the thyroid ultrasound sonography of the SAT patients before they suffered from SAT, we collected age-, sex-, and body mass index (BMI)-matched healthy (without systemic diseases) euthyroid and thyroid autoantibody-negative individuals (the thyroid ultrasonography presented a normal pattern without nodules or with a single nodule <5 mm in diameter) who visited the medical examination center of the hospital for physical examination as control subjects. The thyroid volumes at the time of follow-up of SAT patients were compared with the thyroid volumes of 36 healthy individuals. The clinical data from both groups were recorded and evaluated. The diagnosis of SAT was based on laboratory indicators, such as increased serum levels of FT4 and/or FT3, low serum levels of TSH, increased ESR, and low ^99m^${\text{TcO}}_{4}^{-}$ uptake, or, on ultrasonographic findings, consistent with SAT and clinical features such as thyroid gland tenderness, swelling, and pain without additional inflammatory conditions (8). While they were in the acute phase of SAT, the patients visited the hospital every 15 days. During each visit, the heart rate, body temperature, and ESR were measured. Thyroid function was measured once per month, and thyroid ultrasound was performed once every 6 months. A total of 63 patients successfully completed the 2 years of follow-up visits, while the other 10 patients were lost to follow-up. Patients had high levels of the thyroperoxidase antibody (TPOAb, >50 IU/L) and/or antithyroglobulin antibody (TgAb, >40 IU/L), and typical ultrasound echo patterns of AIT in the background as well were excluded due to the property of painful AIT (9). Then, two patients were ruled out further. Of the 61 patients with complete follow-up data (15 males and 46 females), four became pregnant after the acute stage of SAT and gave birth. All those patients and controls were from Shenyang City, Liaoning Province. Written informed consent was obtained from each patient before their enrolment. Ethical approval was obtained from the Ethics Institutional Review Board of China Medical University prior to subject recruitment.

### Serum Assays

The serum levels of TSH, FT4, and FT3 were measured with electrochemiluminescent immunoassays (Architect i2000SR, Abbott Laboratories, USA). The levels of TPOAb and TgAb were measured by Architect i2000SR (Abbott Laboratories, USA) and Cobas Elecsys 601 (Roche Diagnostics, USA). The ESR was measured using a Micro Test 1 automatic erythrocyte sedimentation analyser (Alifax). The reference ranges provided by the manufacturer were as follows: 0.35–4.94 mIU/L for TSH, 2.63–5.7 pmol/L for FT3, 9.01–19.05 pmol/L for FT4, and 0.00–20 mm/h (female) and 0.00–15 mm/h (male) for the ESR. For the kits from Abbott Laboratories, the reference ranges for TPOAb (0.00–5.61 IU/mL) and TgAb (0.00–4.11 IU/mL) provided by the manufacturer were used. The inter-assay coefficients of variation (CVs) for TPOAb and TGAb were in the range 1.8–3.2% and the total assay CV values were in the range 2.3–4.2%. For Roche Diagnostics, the reference ranges for TPOAb and TgAb were 0.00–34.00 and 0.00–115.00 IU/ml, respectively, as previously described (10, 11). The intra-assay CVs of TPOAb and TGAb were 2.42–5.63 and 1.3–4.9%, respectively. The inter-assay CV values were 5.23–8.16 and 2.1–6.9%, respectively (12). The kits for TPOAb and TgAb from Roche Diagnostics were used only in healthy controls.

### Thyroid Imaging

Sonographic examinations were performed with high-end instruments (HI VISION, Preirus). Most newly diagnosed SAT patients present with echo inhomogeneity and a flaky hypoechoic area located unilaterally or bilaterally in the thyroid lobes along with thyroid pain. The thyroid volume of each patient was measured by ultrasound using Brunn's formula as follows (13): volume = 0.52 (length × width × depth). The value of 0.52 is an approximation of π/6. The length, width, and depth were the longitudinal diameter, the transverse (or lateral) diameter, and the anterior posterior (AP) diameter, respectively. The collective volume was the total volume of the two lobes. The thyroid gland emission computed tomography (ECT) examination was performed with a TruePoint SPECT·CT (Symbia). The scintigraphic picture (^99m^${\text{TcO}}_{4}^{-}$) was checked by means of a thyroid scintiscan. Most SAT patients showed only a small amount of imaging agent in the thyroid gland, and the thyroid images were of poor quality because the thyroid uptake of radioactive ^99m^${\text{TcO}}_{4}^{-}$ was significantly reduced.

### Treatment

Non-steroidal anti-inflammatory drugs (NSAIDs) or glucocorticoids (prednisolone, PSL) were administered to patients with the onset of SAT to relieve clinical symptoms such as fever and pain in the anterior neck. The drug selection and dose used for SAT treatment were based on clinical experience due to the lack of guidelines regarding the selection of PSL and NSAIDs (7). Patients with clinical hypothyroidism within 3 months after the onset of the acute stage of SAT and subclinical hypothyroidism (TSH > 10 mIU/L) 6 months after the onset of the acute stage of SAT were recommended to receive levothyroxine (L-T4) replacement therapy. Patients with mild hypothyroidism (TSH < 10 mIU/L) were treated with L-T4 if they had symptoms of hypothyroidism, were TPOAb-positive, had dyslipidemia, had atherosclerotic diseases, or were pregnant, as recommended in the guidelines for the treatment of hypothyroidism. The initial dose of L-T4 was 12.5 or 25 μg/day. The dose was adjusted according to thyroid function rechecking results after 3–4 weeks. Patients receiving L-T4 treatment, after the thyroid function stabilized for a period of time, tried reducing the dose or discontinuing L-T4 for 1 month; if the TSH value rises again, these patients were considered to have hypothyroidism, at last.

### Statistical Analysis

The professional data processing software SPSS (version 22.0, SPSS Inc.) was used for the statistical analyses. Data with a normal distribution are presented as the mean ± standard deviation (*M* ± SD). Abnormally distributed data are presented as the median (interquartile range). Depending on the data characteristics, Student's *t-*tests, Mann–Whitney *U-*tests, or chi-square tests were used to analyze the differences between groups. Binary logistic regression was performed to analyze the correlations between the initial laboratory indicators and the incidence of hypothyroidism. To assess the risk of hypothyroidism, we performed receiver operating characteristic (ROC) curve analysis based on the early maximum TSH values (within 3 months after SAT onset). A *p* < 0.05 was considered statistically significant.

## Results

### Clinical Features

The basic clinical characteristics of the 61 SAT patients are shown in Tables 1, 2. In the initial stage of SAT, most of the patients (81.4%) visited a physician because of anterior neck pain, and 25.4% had body temperatures higher than 38°C. Palpation revealed thyroid enlargement in 95.8% of the patients. Possibly due to excessive thyroid hormone release, some patients also experienced increased heart rates; the heart rates of 28.2% of the patients were faster than 100 bpm.

**Table 1:** Clinical and demographic characteristics of patients with subacute thyroiditis (SAT) and healthy controls.

**Characteristics**	**Healthy controls (*n* = 36)**	**SAT (*n* = 61)**	***P*-values**
Gender (female/male)	30/6	46/15	0.360
Age (mean ± s.d., years)	44.06 ± 9.88	44.07 ± 9.20	0.996
BMI (mean ± s.d., kg/m2)	23.78 ± 3.26	23.71 ± 3.29	0.924
TSH (median, mIU/L)	1.725 (1.213–2.495)	0.015 (0.005–0.230)	<0.001
FT_3_ (median, pmol/L)	4.16 (3.96–4.57)	7.05 (5.47–9.22)	<0.001
FT_4_ (median, pmol/L)	15.32 (13.94–16.87)	21.16 (15.77–28.58)	<0.001
TPOAb (median, IU/mL)	5.00 (5.00–5.00)^a^	1.03 (0.46–8.66)^b^	Not done
TgAb (median, IU/mL)	11.25 (10.04–13.26)^a^	7.80 (2.58–23.49)^b^	Not done

a *Roche Diagnostics*.

b *Abbott Laboratories*.

**Table 2:** Clinical features of subacute thyroiditis (SAT).

**Characteristics**	
*Initial*	
Anterior neck pain (%)	81.4 (48/59)
Temperature (above 38 degrees) (%)	25.4 (15/59)
Palpation (thyroid enlargement) (%)	95.8 (46/48)
Heart rate (above 100 beats/min) (%)	28.2 (11/39)
ESR (mean ± s.d., mmH_2_O)	56.8 ± 28.15
ESR (above 100 mmH_2_O) (%)	18.6 (11/59)
ECT for poor imaging (%): Bilateral Unilateral	92.6 (25/27) 3.7 (1/27)
Not regular	3.7 (1/27)
Therapy (%): NSAIDs	75.4 (46/61)
NSAIDs and corticosteroids	11.5 (7/61)
Corticosteroids	9.8 (6/61)
No medication	3.3 (2/61)
*Prognosis*	
1 year's hypothyroidism (%)	45.8 (27/59)
2 years' hypothyroidism (%)	32.8 (20/61)

*ESR, erythrocyte sedimentation rate; ECT, Emission Computed Tomography; NSAIDs, Non-steroidal anti-inflammatory drugs*.

### Laboratory and Imaging Data

The primary ESR levels were elevated in most SAT patients, and 18.6% of the patients had ESR values >100 mm/h (Table 2). The ESR levels recovered to the normal range within 3 months in 83.0% (44/53) of patients. Ultrasound examination revealed that hypoechogenic areas existed in 98.3% of the patients at the onset of acute SAT (Table 3). Among the SAT patients who underwent ECT examinations (*n* = 27), 25 showed a reduced distribution of the imaging agent in both thyroid lobes. In Table 3, we listed the variable changes during the initial follow-up and at 6 months, 1 year, and 2 years after SAT. At the onset of SAT, we found that the percentages of TPOAb- and TgAb-positive patients had increased by 18.0 and 49.2%, respectively. At the final follow-up, the rates of antibodies gradually decreased to 1.8 and 12.3%. The incidence of thyroid nodules did not change significantly during the follow-up period. However, five patients continued to have hypoechoic regions in the thyroid gland 2 years after SAT onset.

**Table 3:** Variable changes during follow-up.

**Characteristics**	**Time point**			
	**Initial**	**6 month**	**1 year**	**2 year**
FT3 (pmol/L)	7.1 (5.5–9.2), *n* = 61	4.5 ± 0.6, *n* = 51	4.6 ± 0.6, *n* = 54	4.6 (4.2–5.0), *n* = 57
FT4 (pmol/L)	21.2(15.8–28.6), *n* = 61	12.7 (11.7–14.8), *n* = 52	14.0 ± 3.1, *n* = 54	15.3 ± 2.4, *n* = 57
TSH (mIU/L)	0.015 (0.005–0.23), *n* = 61	3.2 (2.2–4.5), *n* = 52	3.9 ± 2.3, *n* = 54	3.6 ± 1.8, *n* = 57
TPOAb positive (%)	18.0 (11/61)	20.4 (10/49)	11.5 (6/52)	1.8 (1/57)
TgAb positive (%)	49.2(30/61)	24.5 (12/49)	19.2 (10/52)	12.3 (7/57)
Hypoechogenic (%)	98.3(59/60)	43.8 (21/48)	23.9 (11/46)	9.4 (5/53)
Thyroid nodules (%)	41.7 (25/60)	40.0 (19/48)	34.0 (16/47)	35.2 (19/54)

### Therapy

The medications administered for the early relief of symptoms and signs in the 61 SAT patients were as follows. Forty-six patients were administered NSAIDs, six patients were treated with PSL therapy, seven were prescribed a combination of NSAIDs and PSL therapy, and two patients did not receive any medication. We found that only 1 out of 13 patients on PSL therapy had hypothyroidism at 2 years; however, 19 of 48 patients who did not receive PSL had hypothyroidism (*p* = 0.066).

### Thyroid Function

At the 1 and 2 years follow-up time points, the TSH values of patients without L-T4 treatment exceeded the normal reference values considered to indicate hypothyroidism. For patients treated with L-T4, if the TSH value increased after reducing the dose or discontinuing L-T4 for 1 month, these patients were considered to have hypothyroidism. During the 2 years follow-up period, the incidences of hypothyroidism at 1 and 2 years were 45.8 and 32.8%, respectively (Table 2). Seventeen of the 20 hypothyroid patients received L-T4 therapy. Their final TSH level at the end of the 2 years follow-up was 5.03 ± 2.34 mIU/L, significantly higher than that of SAT patients without hypothyroidism (2.82 ± 1.09 mIU/L, *p* < 0.001). We then evaluated the relationship between early clinical indicators and hypothyroidism at 2 years (Table 4A) and performed a binary logistic regression analysis to assess the early clinical indicators that can predict the development of hypothyroidism (Table 4B). The results revealed that the early maximum TSH value (within 3 months after SAT onset) was closely related to the incidence of hypothyroidism at 2 years. The OR value was 1.18 (95% CI = 1.01–1.38, *p* = 0.032).

**Table 4A:** Patient characteristics.

**Patients characteristics**	**At 2 year**		***P*-value**
	**Euthyroidism (*n* = 41)**	**Hypothyroidism (*n* = 20)**	
Sex (female, %)	73.2%	80%	0.791
Treatment method (PSL, %)	29.3%	5%	0.066
Age (year)	43.7 ± 9.1	44.8 ± 9.7	0.695
	43.78		
Initial levels of FT3 (pmol/L)	7.6 ± 2.9	7.3 (5.8–10.1)	0.419
Initial levels of FT4 (pmol/L)	22.6 ± 8.0	23.4 (16.0–30.5)	0.510
Initial levels of TSH (mIU/L)	0.014 (0.004–0.170)	0.020 (0.005–0.604)	0.424
Initial levels of TPOAb (IU/mL)	1.2 (0.5–9.3)	0.6 (0.5–8.4)	0.901
Initial levels of TgAb (IU/mL)	7.8 (2.4–22.2)	5.4 (2.6–23.5)	0.752
Maximal FT3^b^ (pmol/L)	7.7 (6.0–10.5)	9.3 ± 4.1	0.500
Maximal FT4^b^ (pmol/L)	25.3 ± 8.7	27.6(21.0–33.2)	0.293
Minimal TSH^b^ (mIU/L)	0.009 (0.003–0.043)	0.005 (0.004–0.173)	0.933
Maximal TSH^b^ (mIU/L)	4.5 (2.6–7.7)	17.1 (8.3–70.5)	0.000
Maximal ESR^b^ (mmH2O)	54.5 (44.0–77.3)	58.0 ± 32.2	0.661
ESR recovery time (day)	57.4 ± 39.5	59.1 ± 48.1	0.893

**Table 4B:** Results of univariate and multivariate regression analysis of risk factors for hypothyroidism versus euthyroidism at 2 year after the onset of SAT.

**Variables entered as covariates**	**Hypothyroidism vs. euthyroidism at 2 year**			
	**Univariate**		**Multivariate**	
	**OR(95%CI)**	***P*-value**	**OR(95% CI)**	***P*-value**
Treatment method^a^	0.08 (0.01–0.77)	0.028	0.13 (0.01–1.51)	0.134
Maximal FT4^b^	1.03 (0.98–1.09)	0.214	1.03 (0.97–1.10)	0.300
Maximal TSH^b^	1.18 (1.04–1.34)	0.012	1.18 (1.01–1.38)	0.032

a *The treatment is divided into prednisolone and no prednisolone*.

b *Within 3 months of SAT onset*.

### ROC Analysis of the TSH Value

**Figure 1:**
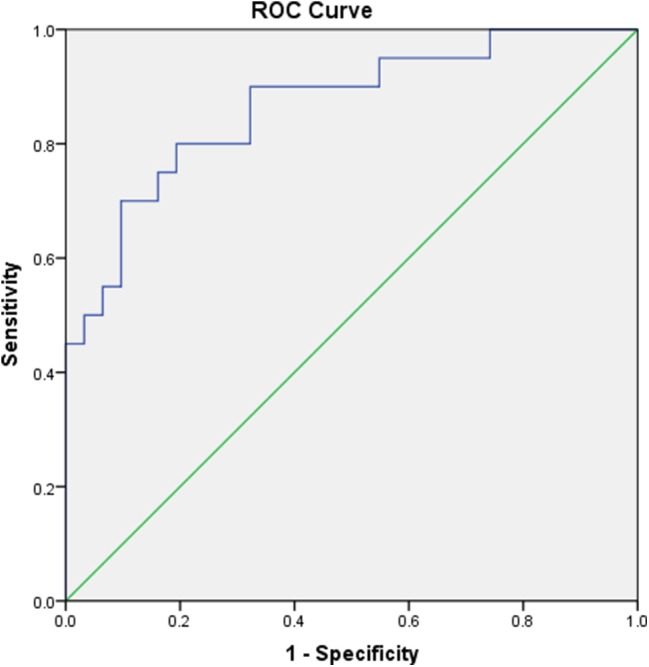
Receiver operating characteristic (ROC) curves assessing the risk of hypothyroidism 2 years after the onset of SAT based on the early maximum TSH value.

ROC analysis was performed to assess the 2 years risk of hypothyroidism based on the early maximum TSH value. The early maximum TSH value showed a predictive value, with an area under the ROC curve (AUC) of 0.866 for hypothyroidism after 2 years of follow-up *vs*. euthyroidism (*p* < 0.001; Figure 1). According to Youden's index, the best cutoff value for the TSH level was 7.83 μIU/L. The sensitivity and specificity were 80.0 and 80.6%, respectively.

### Thyroid Volume

**Figure 2:**
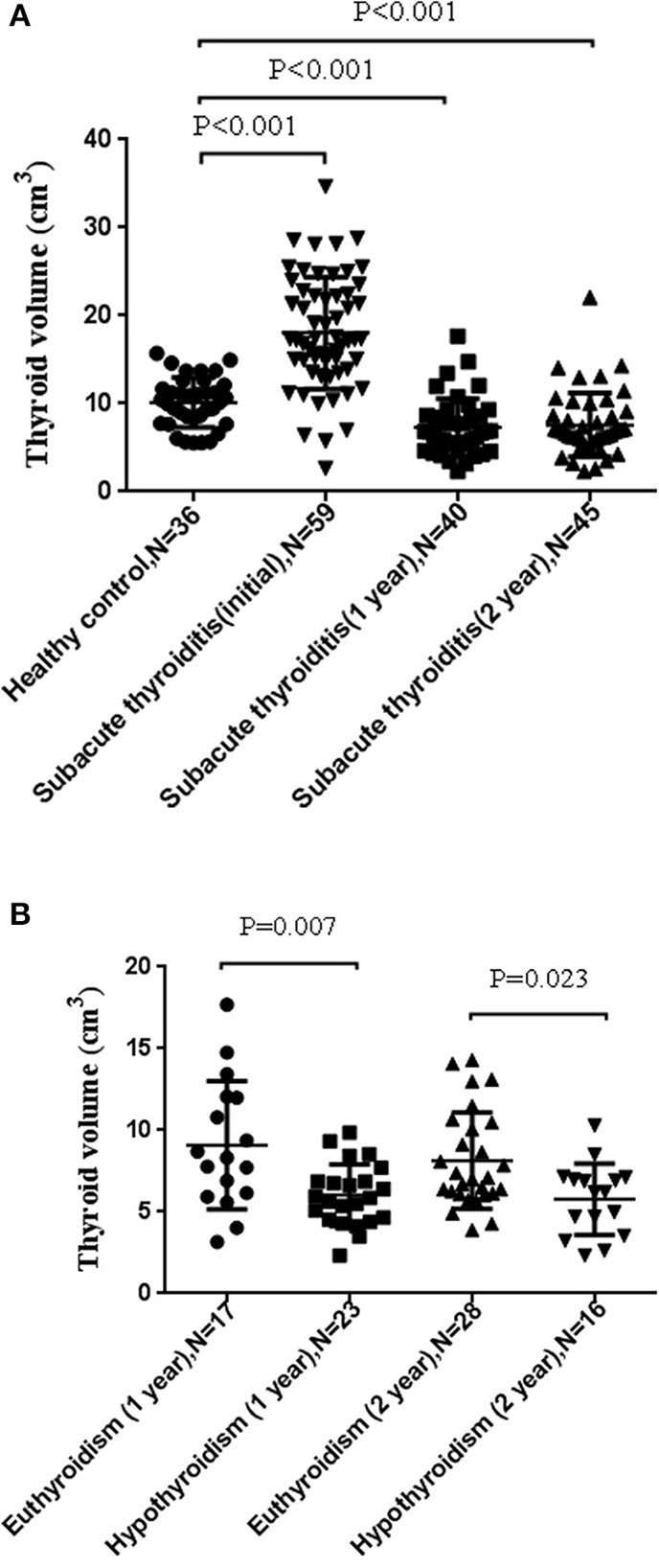
The thyroid volume of subacute thyroiditis (SAT) at 1 and 2 years was significantly smaller. **(A)** Comparison of the thyroid gland volumes between SAT patients at initial, 1 and 2 years of follow-up and those of healthy controls. **(B)** Comparison of thyroid volumes of SAT patients after 1 year and 2 years of hypothyroidism with those of euthyroid individuals.

We wanted to further explore the effect of suffering SAT on the thyroid volume. Since the thyroid volumes of patients with pre-onset of SAT were not available, we randomly enrolled individuals with normal thyroid function and without obvious abnormality in ultrasonic performance, who are thyroid autoantibody-negative, and without other systemic diseases as the normal thyroid volume control group (Table 1). The thyroid volume within 3 months after SAT onset was regarded as the initial thyroid volume. We compared the thyroid gland volumes at 1 or 2 years follow-up of SAT patients (four patients becoming pregnant during the follow-up period were excluded since pregnancy may affect the thyroid volume) with those of the control group and the initial thyroid volumes. We found that the volumes of the thyroid glands of SAT patients (1 year, 6.70 (5.16–8.66) cm^3^; 2 years, 6.88 (5.69–8.89) cm^3^) were significantly smaller than those of the healthy control group (10.13 ± 2.82 cm^3^, *p* < 0.001). In addition, the initial thyroid volumes significantly increased compared with the normal control group (initial 18.04 ± 6.36 *vs*. control 10.13 ± 2.82 cm^3^, *p* < 0.001) (Figure 2A). No significant difference was found in the thyroid gland volumes between SAT patients at the 1 and 2 years follow-up periods. The thyroid volume [7.05 (6.09–10.38) cm^3^] at the 2 years follow-up of SAT patients without hypothyroidism was still lower than that of the normal controls (*p* = 0.021). Furthermore, the thyroid volumes of SAT patients with hypothyroidism either at 1 year (6.03 ± 1.89 cm^3^) or 2 years (5.78 ± 2.10 cm^3^) follow-up were significantly smaller than those of euthyroid SAT patients (*p* = 0.007 and *p* = 0.023; Figure 2B).

## Discussion

The present study showed that the early maximum TSH value (within 3 months after onset) at the acute onset of SAT could be used as an effective indicator of the risk of hypothyroidism 2 years after the onset of SAT. Compared with healthy controls, the thyroid volumes of patients increased significantly after the onset of SAT, while at 1 and 2 years these were significantly smaller, and the decrease in thyroid volume over that 2 years period observed in patients with hypothyroidism was greater than that in euthyroid patients.

Researchers consider SAT to be the result of a viral infection rather than a bacterial infection (14, 15), with no epidemiological evidence regarding the nature of the infection among populations (16). Sato et al. (7) noted that the symptoms of the PSL group disappeared significantly faster than those of the NSAID group, while in our study, no significant difference in ESR recovery time was found between patients who used corticosteroids and those who did not. Endocrinologists should pay attention not only to short-term symptom improvement but also to the long-term risk of hypothyroidism caused by SAT. Early transient hypothyroidism is common in patients with SAT, but permanent hypothyroidism is relatively rare, and reports have shown that permanent hypothyroidism develops in 5.9–15% of SAT patients during the follow-up period (6, 17, 18). From our analysis of 2 years of follow-up, the incidence rates of hypothyroidism after 1 and 2 years were 45.8 and 32.8%, respectively. Perhaps due to the short follow-up period, the incidences of hypothyroidism were relatively high. In addition, the 10 patients who were lost to follow-up were likely to have normal thyroid function after the acute stage of SAT, so the 1 and 2 years incidence rates of hypothyroidism in our study may actually be lower than those reported. Nishihara et al. (17) reported that compared with anti-inflammatory treatment, PSL treatment was more likely to result in normal thyroid function in patients who were followed for over 6 months. However, in the 28 years follow-up period in the study by Fatourechi et al. (6), corticosteroid therapy was found to alleviate the acute symptoms during the onset of SAT, but could not prevent early or delayed hypothyroidism. Fatourechi et al. (6) noted that, although no significant difference could be found within the first 6–12 months after SAT onset, compared with patients who receive PSL treatment, patients who use corticosteroids are more likely to have hypothyroidism at 1 year (6). The variation in the results of the above studies may be due to the differences in sample size and follow-up duration. In the present study, we found that PSL therapy could reduce the incidence of hypothyroidism, but the difference was not significant in the incidence of hypothyroidism between the PSL group and the NSAID group (*p* = 0.066). This may be due to the insufficient number of patients, especially the small number of patients receiving prednisolone. A randomized controlled study of SAT patients can help to determine whether glucocorticoid therapy can reduce the incidence of hypothyroidism compared with NSAIDs.

Some scholars have noted that there is no correlation between the development of hypothyroidism and the levels of thyroid hormones or inflammatory indicators in laboratory examinations at SAT onset and that thyroid dysfunction is not correlated with changes in antibody titers (18, 19). However, our binary logistic analysis and ROC curve analysis results revealed that the early maximum TSH value was closely related to the incidence of hypothyroidism at 1 and 2 years after the onset of SAT. According to Youden's index, the best cutoff value for the TSH level was 7.83 mIU/L, and the sensitivity and specificity were 80.0 and 80.6%, respectively. We suggest that an early maximum TSH value of 7.83 mIU/L could be used as an effective indicator of the risk of hypothyroidism in SAT patients.

In addition, we investigated the thyroid gland volume in SAT patients. We found that the thyroid volumes of patients increased significantly after the onset of SAT, while the thyroid volumes at 1 and 2 years were significantly smaller than those of age- and sex-matched healthy controls in our 2 years of follow-up, which was consistent with the results of the study by Benker et al. (20) that was conducted a relatively long time ago. It makes sense that, at diagnosis (or within 3 months from it), the thyroid volume was increased by inflammation and that, at 1 and 2 years, the volumes were finally reduced due to the initial damage that had occurred and the inflammation having disappeared. We also found that even if there was no hypothyroidism left in the end, the thyroid volumes of patients who suffered SAT were still significantly smaller than those of the healthy controls. Furthermore, among SAT patients, hypothyroid SAT patients had much smaller thyroid volumes than euthyroid SAT patients at 1 or 2 years follow-up. And this reduction in thyroid volume was not due to the suppression of TSH by L-T4 therapy.

The limitations of the present study are as follows. The thyroid volumes of patients before they suffered from SAT were not available. However, due to the fact that the patients were not enrolled at the same time after the onset of SAT, we took the thyroid volume within 3 months after SAT onset as the initial thyroid volume. The follow-up period was relatively short, and the sample size needs to be larger in future studies. Additionally, although all the patients from the same area have adequate iodine intake (11), we did not investigate the iodine intake and the urinary iodine concentration of each individual, which may have an impact on antibody positivity and thyroid nodules and volumes.

In conclusion, during the 2 years of follow-up in this study, we found that the thyroid gland volumes of SAT patients, especially those with hypothyroidism, were smaller than those of healthy controls after the acute stage of the disease. The higher early maximum TSH value within 3 months after SAT onset may be the risk factor for the incidence of hypothyroidism 2 years later.

## Data Availability Statement

The datasets generated for this study are available on request to the corresponding author.

## Ethics Statement

The studies involving human participants were reviewed and approved by Ethics Institutional Review Board of China Medical University. The patients/participants provided their written informed consent to participate in this study.

## Author Contributions

NZ, Y-SL, Z-YS, and W-PT contributed to the concept and design of this study. Y-SL reviewed the final manuscript. NZ, SW, X-JC, M-SH, and S-WW organized the database and statistical analysis. Y-GL, LZ, and W-NW acquired sonographic data. NZ wrote the first draft of the manuscript. All authors contributed to the manuscript refinement and approved the submitted version.

### Conflict of Interest

The authors declare that the research was conducted in the absence of any commercial or financial relationships that could be construed as a potential conflict of interest.
